# Case of Lacerated Wound of the Thorax

**Published:** 1839-02-02

**Authors:** George Tomlinson

**Affiliations:** Roadstown, N. Jersey


### *Case of Lacerated Wound of the Thorax.* By George Tomlinson, M. D.

On the second of October, 1838, I was called to see E. D., aged twenty-three, a house carpenter, who, while standing on the roof of a house, a story and a half high, putting weather-boards on a two story part adjoining, fell on a pale fence, (a few feet from the house,) in such a manner as to bring the left arm on one side, while his body went on the opposite side of the fence; one of the pales penetrating the axilla, and passing upward, in a somewhat anterior direction, escaping the axillary plexus, and entering the chest just above the first rib.

An hour had elapsed from the receipt of the injury before I arrived, and there had been but very little hæmorrhage from the wound. No expectoration, and but little inclination to cough; the countenance very pale, and the pulse considerably depressed, dyspnœa, was obliged to have his shoulders very much elevated. On raising the arm, I found the air passed readily through the wound, in and out of the chest. Wishing to avoid this, as much as possible, I made no further examination than was necessary to remove a leaf which had been carried into the wound, from a peach tree which hung over the fence; after which, I dressed it with adhesive plaster, and gave an opiate.

In the evening I saw him again. Pulse more full; dyspnœa very distressing. The rattling of the air in the chest could be distinctly heard, and, to use his own expression, he was "obliged to change his position, frequently, to remove the wind, or he could not breathe."

3*d.* Less difficulty of breathing, pulse natural—gave a laxative.

4*th.* Better.

5*th.* Dressed the wound; the internal edges had healed.

6*th.* Four days after the accident, was removed home, four miles, without any injury.

He could not lie in a recumbent posture for ten or twelve days. The regimen, for the first few days, was strictly antiphlogistic. No symptoms of inflammation occurred, and in a few weeks the wound was entirely healed, leaving no unpleasant consequences whatever.

*Roadstown, N. Jersey, Jan. 24th,* 1839.

